# Supplementary material for: auts2 Features and Expression Are Highly Conserved during Evolution Despite Different Evolutionary Fates Following Whole Genome Duplication
Source: Cells. 2022 Aug 30;11(17):2694. doi: 10.3390/cells11172694 (PMC9454499; doi:10.3390/cells11172694)
Supplement: Supplementary file 1 [file cells-11-02694-s001.zip › Figure S3.pdf]

```
auts2b_D.rerio_exon9             KPGKWCAMHVHIAWQVYHHQQKIK
A.anguilla_12:5647264-5647247    --------HVHICW----------
C.harengus_NW_012221820.1:1032   RPRGWYAMHVTIAWQVYLQQQKAK
O.latipes_13:12365425-12369417   --GKGGAAHVHIAWGIYFHKQ---
S.maximus_3:11933378-11933316    KRGRLGALHVCIAWKIYYHKQ---
X.maculatus_18:25652475-256525   -------LHVYIAWRIYYHKQ---
G.aculeatus_groupI:10844121-10   KRVRWSALHVGIAWRIYYHKQ---
T.nigroviridis_16:2661543-2661   ---RWGALHVRIAWKIYHHKQ---
O.niloticus_LG14:25797068-2579   RRGRWGALHVCIAWKIYYHKQ---
A.percula_9:2132141-2132197      --GRWGALHVCIAWKIHYHKQ---


auts2a_P.nattereri_exon7         KPGKWCAMHVHIAWQIYHHQQKGK
A.mexicanus_20:12208335-122083   -------IHKHICWQ---------

auts2a_E.electricus_exon10       KPGKWCAMHVHIAWQIYHHQQKGK
P.hypophthalmus_17:8831365-883   --------HNHICW----------
I.punctatus_16:18136191-181362   --------HVHITWN---------
I.punctatus_16:21823263-191704   --------HVHINWN---------
I.punctatus_KV453361.1:77808-7   ---------VHIAW----------
I.punctatus_KV453361.1:84208-8   --------------QIYHH-----
```
