# Supplementary material for: auts2 Features and Expression Are Highly Conserved during Evolution Despite Different Evolutionary Fates Following Whole Genome Duplication
Source: Cells. 2022 Aug 30;11(17):2694. doi: 10.3390/cells11172694 (PMC9454499; doi:10.3390/cells11172694)
Supplement: Supplementary file 1 [file cells-11-02694-s001.zip › Figure S4.pdf]

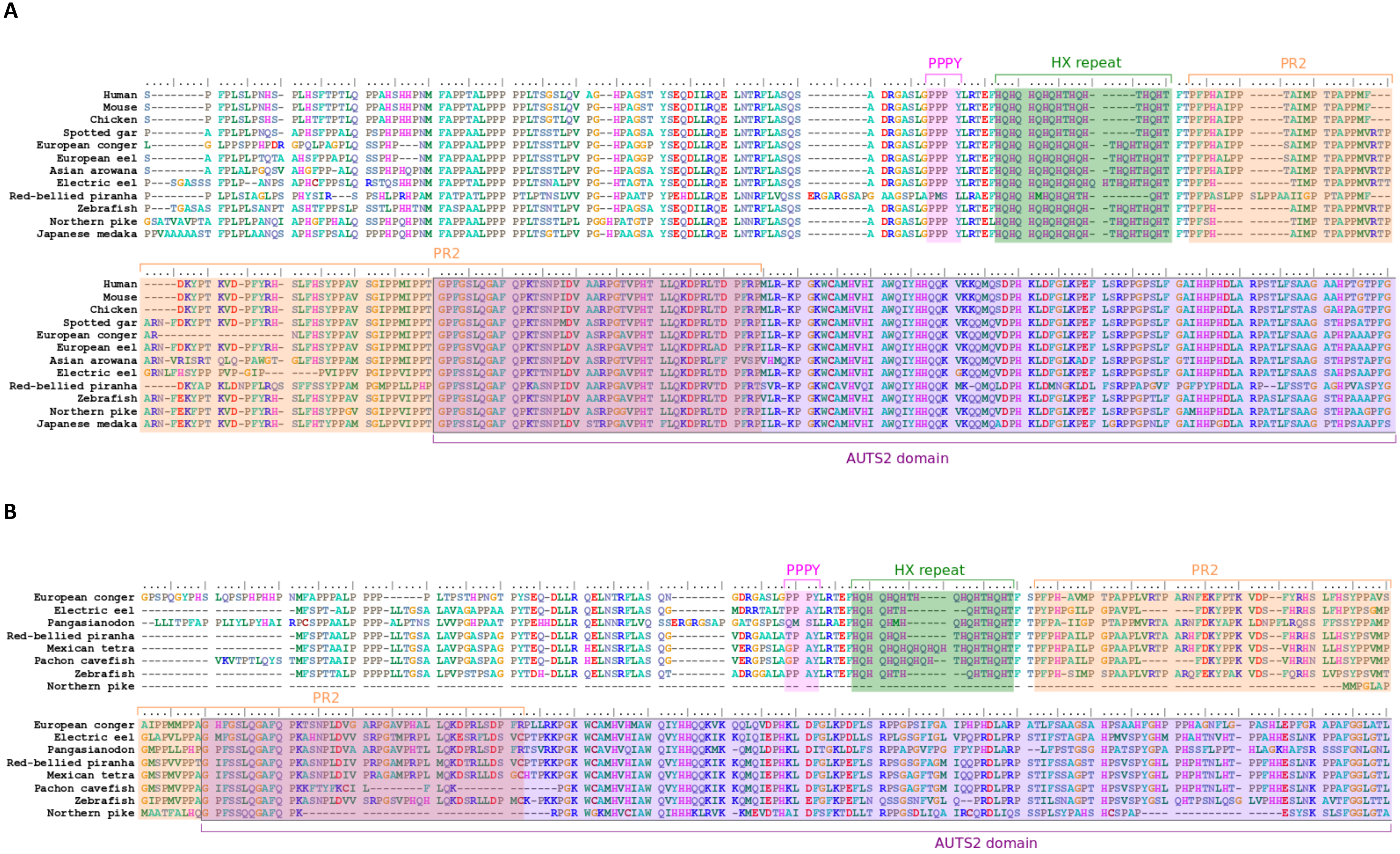
A
PPPY
HX repeat
PR2
Human
Mouse
Chicken
Spotted gar
European conger
European eel
Asian arowana
Electric eel
Red-bellied piranha
Zebrafish
Northern pike
Japanese medaka
AUTS2 domain
B
PPPY
HX repeat
PR2
European conger
Electric eel
Pangasianodon
Red-bellied piranha
Mexican tetra
Pachon cavefish
Zebrafish
Northern pike
AUTS2 domain
