# Supplementary figures and images for: auts2 Features and Expression Are Highly Conserved during Evolution Despite Different Evolutionary Fates Following Whole Genome Duplication
Source: Cells. 2022 Aug 30;11(17):2694. doi: 10.3390/cells11172694 (PMC9454499; doi:10.3390/cells11172694)

A

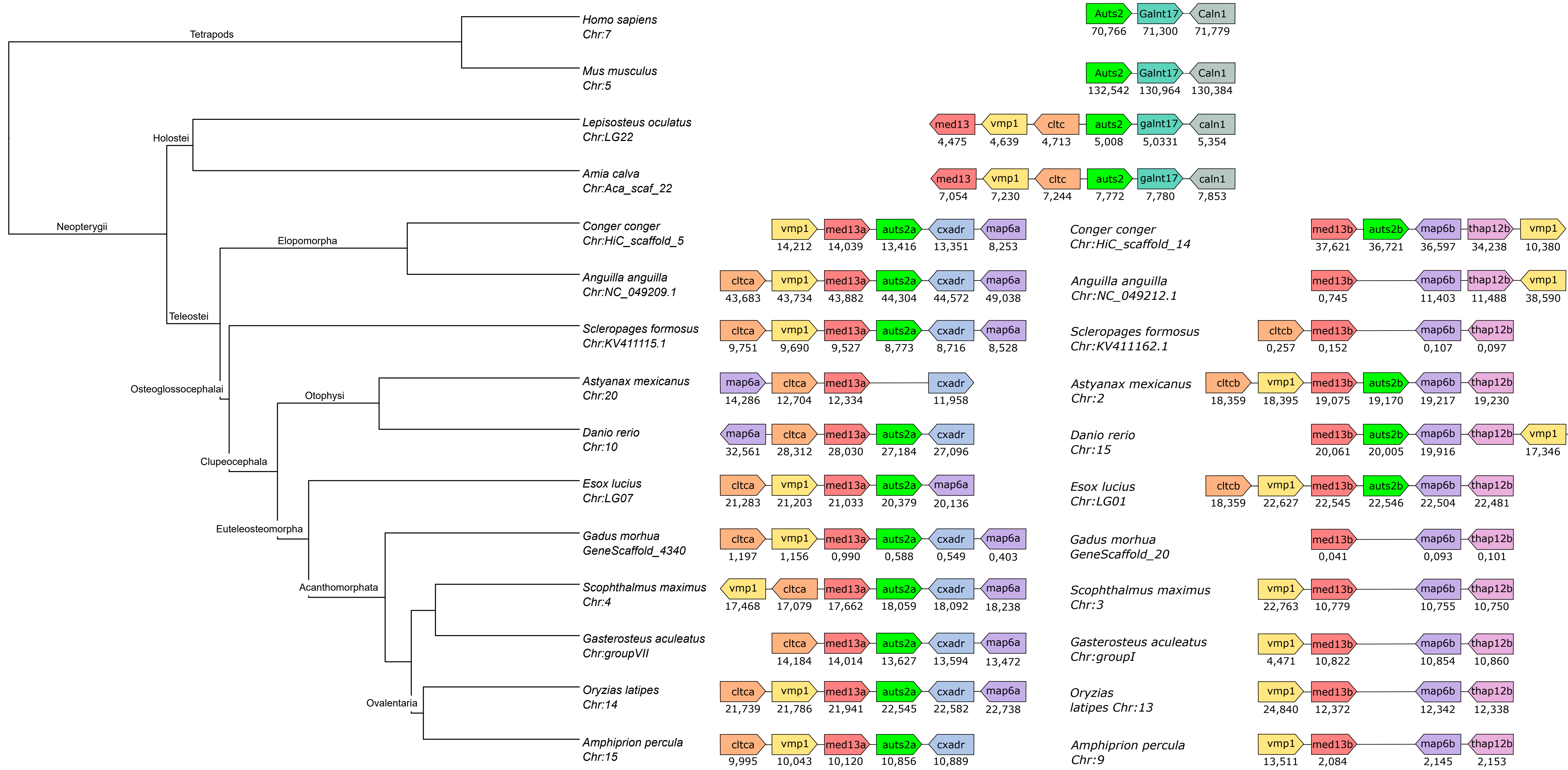

B

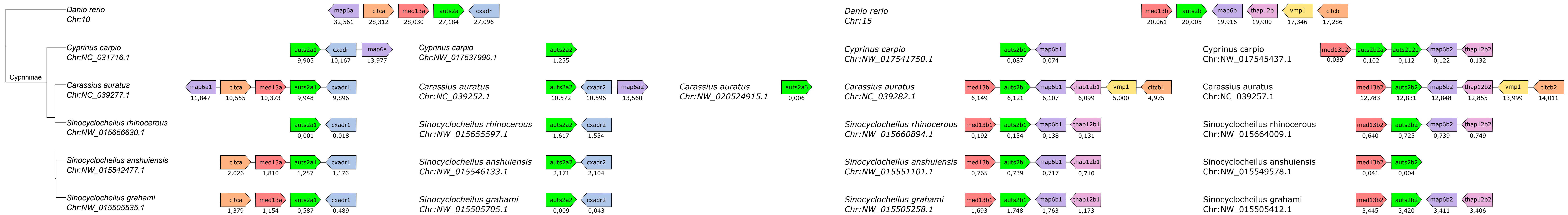

C

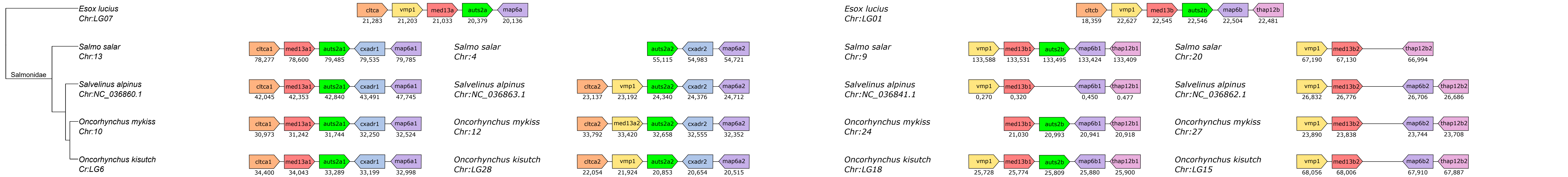

Supplement: Supplementary file 1 [file cells-11-02694-s001.zip › Figure S1.pdf]

A

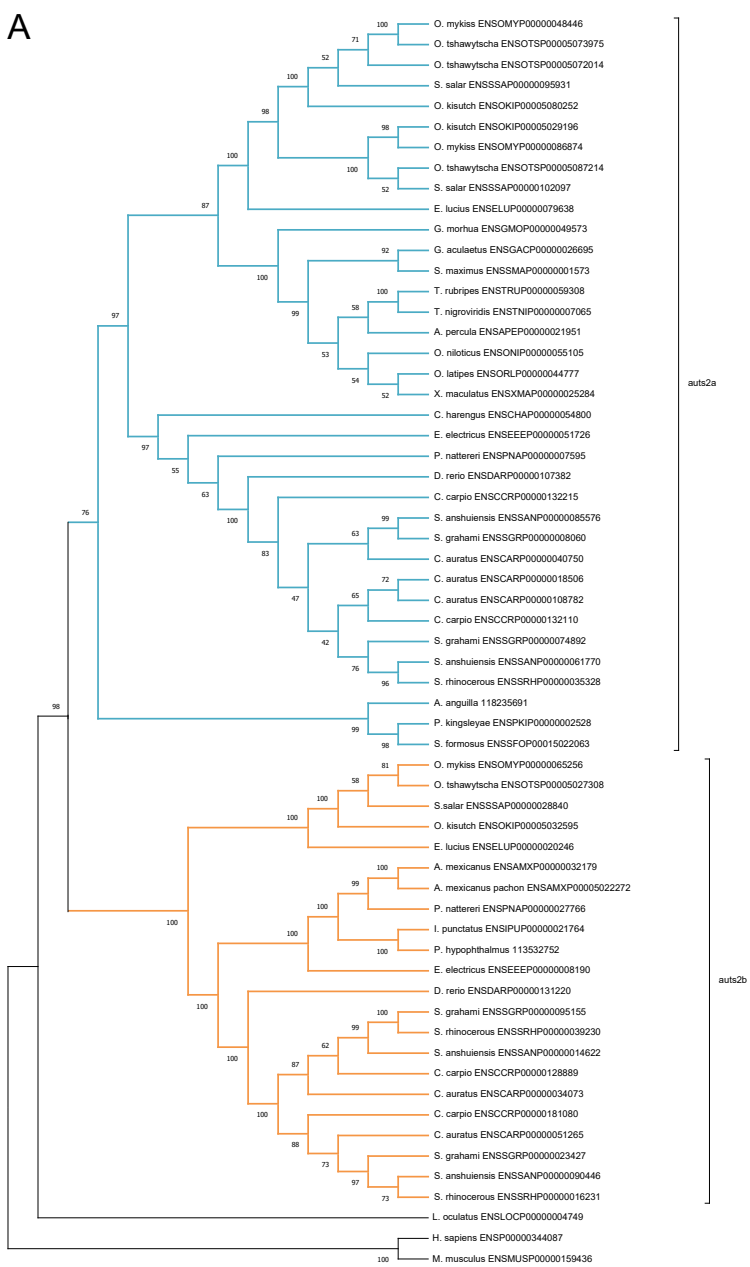

B

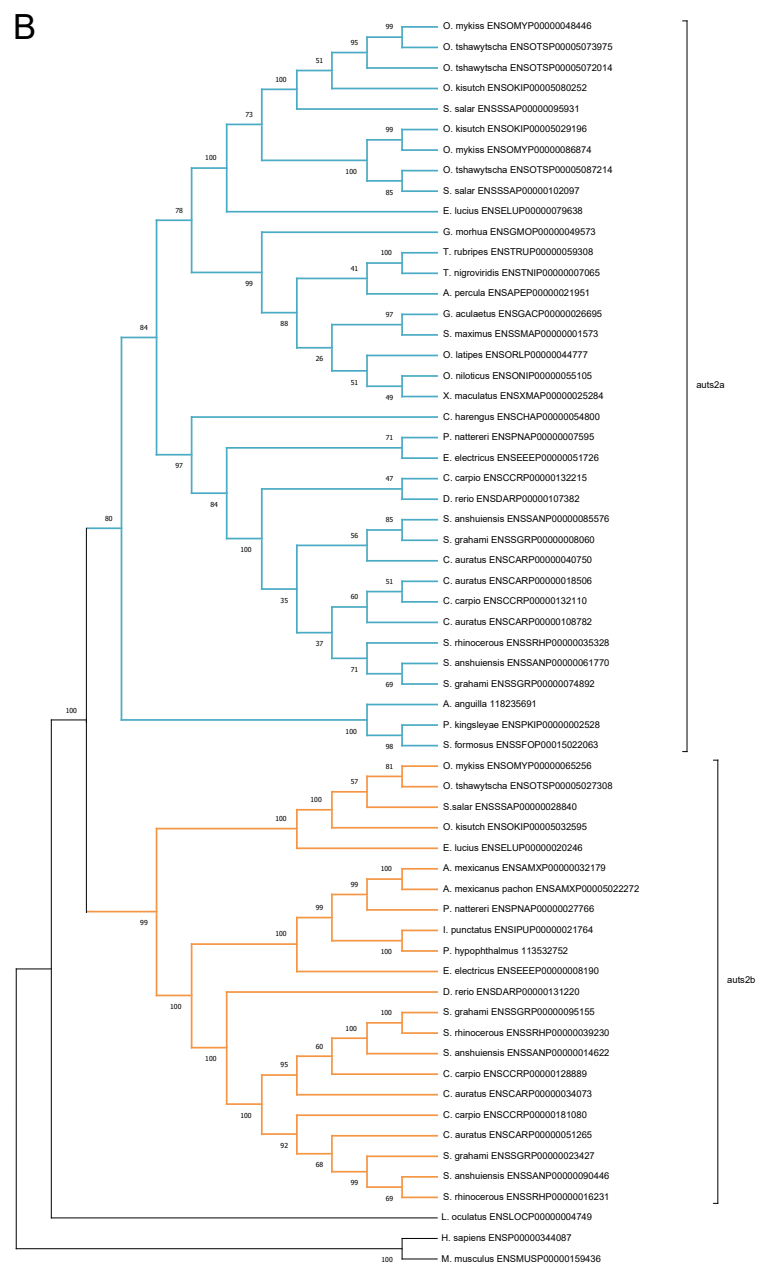

Supplement: Supplementary file 1 [file cells-11-02694-s001.zip › Figure S2.pdf]
